# Systemic inflammation and acute kidney injury after colorectal surgery

**DOI:** 10.1186/s12882-024-03526-w

**Published:** 2024-03-11

**Authors:** John D. Mannion, Assar Rather, Adrianne Fisher, Kelly Gardner, Nessreen Ghanem, Sheila Dirocco, Gary Siegelman

**Affiliations:** Bayhealth Medical Center, Dover, DE United Kingdom

**Keywords:** Acute kidney injury, Aseptic inflammation, Complications

## Abstract

**Background:**

In this retrospective review, the relative importance of systemic inflammation among other causes of acute kidney injury (AKI) was investigated in 1224 consecutive colorectal surgery patients. A potential benefit from reducing excessive postoperative inflammation on AKI might then be estimated.

**Methods:**

AKI was determined using the Kidney Disease Improving Global Outcomes (KDIGO) criteria. The entire population (mixed group), composed of patients with or without sepsis, and a subpopulation of patients without sepsis (aseptic group) were examined. Markers indicative of inflammation were procedure duration, the first postoperative white blood cell (POD # 1 WBC) for the mixed population, and the neutrophil-to-lymphocyte ratio (POD #1 NLR) for the aseptic population. Multivariable logistic regression was then performed using significant (*P* < 0.05) predictors. The importance of inflammation among independent predictors of AKI and AKI-related complications was then assessed.

**Results:**

AKI occurred in 24.6% of the total population. For the mixed population, there was a link between inflammation (POD # 1 WBC) and AKI (*P* = 0.0001), on univariate regression. Medications with anti-inflammatory properties reduced AKI: ketorolac (*P* = 0.047) and steroids (*P* = 0.038). Similarly, in an aseptic population, inflammation (POD # 1 NLR) contributed significantly to AKI (*P* = 0.000). On multivariable analysis for the mixed and aseptic population, the POD #1 WBC and the POD #1 NLR were independently associated with AKI (*P* = 0.000, *P* = 0.022), as was procedure duration (*P* < 0.0001, *P* < 0.0001). Inflammation-related parameters were the most significant contributors to AKI. AKI correlated with complications: postoperative infections (*P* = 0.016), chronic renal insufficiency (CRI, *P* < 0.0001), non-infectious complications (*P* = 0.010), 30-day readmissions (*P* = 0.001), and length of stay (LOS, *P* < 0.0001). Inflammation, in patients with or without sepsis, was similarly a predictor of complications: postoperative infections (*P* = 0.002, *P* = 0.008), in-hospital complications (*P* = 0.000, *P* = 0.002), 30-day readmissions (*P* = 0.012, *P* = 0.371), and LOS (*P* < 0.0001, *P* = 0.006), respectively.

**Conclusions:**

Systemic inflammation is an important cause of AKI. Limiting early postsurgical inflammation has the potential to improve postoperative outcomes.

## Introduction

Acute kidney injury is associated with significant perioperative and medium-term morbidity and mortality [1]. The etiologies of acute kidney injury (AKI) are diverse and multiple; perioperative hypotension [2], the amount [3, 4] and composition of fluid administration [5], postoperative hyperglycemia [6] and nephrotoxic medications [7] are some of the most studied risk factors.

There is some evidence that these risk factors are modifiable: application of the Kidney Disease Improving Global Outcomes (KDIGO) Care Bundle prevented the development of AKI in both the cardiac [8] and general surgery populations [9]. Avoiding chloride-rich fluids [10] and the maintenance of euvolemia, rather than the promotion of restrictive or excessively liberal fluids lower the incidence of AKI [3]. In addition, avoidance of nephrotoxic medications is beneficial [8].

Systemic inflammation exacerbates local kidney damage from other causes and may induce a prolonged inflammatory state affecting multiple organ systems [11, 12]. Further, because colon surgery is often needed in the presence of infection, both septic and aseptic inflammation must be considered. In this study, we examined the multiple causes of AKI after colorectal surgery, to determine the relative contribution of aseptic systemic inflammation, and assess the potential for reduction in AKI with the modification of inflammation.

## Methods

The electronic records of 1224 consecutive patients undergoing surgery for colonic resection or stoma procedures from August 1, 2016, until December 31, 2022, at Bayhealth Medical Center (Dover, DE) were reviewed retrospectively. The database includes elective and emergent procedures, and open or minimally invasive techniques, with no exclusions for age or comorbidities. A colorectal specialist and general surgeons performed the surgery. In 2017 an early recovery after surgery program was established for elective cases. The cases were managed as previously described [13]. The study was approved by the Bayhealth Institutional Review Board.

### Primary outcome

The primary outcome of interest was the relative contribution of systemic inflammation to acute kidney injury and AKI-related complications.

### Definition of AKI and groups examined

AKI was defined using the KDIGO criteria, which included any creatinine increase of ≥ 0.3 mg/dl within the first 48 h after surgery, or a creatinine that was ≥ 1.5 times the baseline creatinine from day 3 to 7 [14]. Urine output was not used to determine AKI. The WBC on POD #1 and its derivatives were considered markers of inflammation. Postoperative day # 1 interleukin-6 levels correlated with the duration and invasiveness of surgery, as shown by Neff et al., so we also included procedure duration as a measure of inflammation [15].

The groups examined included the total population of 1224 patients, which included 1068 patients without concomitant infection, and 156 patients with infection on the day of surgery. This group was called the mixed population. A subgroup of patients without any concomitant infection (aseptic group) was also examined.

Any patient with a positive culture on the day of surgery was considered to have a preoperative infection; any patient with a positive culture from Day 1 through Day 30 was considered to have a postoperative infection. Any postoperative inflammation in patients with active infection was the combined result of the septic process and surgical dissection; the immediate postoperative inflammation in non-septic patients was considered aseptic inflammation.

### Predictors of AKI

Previously reported causes of AKI were examined for this patient population, with an interest in causes related to inflammation. The predictors of AKI were extracted from five broad categories: demographic information, preoperative laboratories, peri-procedural laboratories, procedure-related characteristics, and medications. The following are explanations for the choice of the selected predictors.

### Demographic predictors

Many predictors, such as American Society of Anesthesia (ASA) scores, body mass index (BMI), and sex were chosen because of their demonstrated importance in the literature. For demographic information, the age was categorized as elderly for sixty and greater. Diabetics were considered as those with a clinical history of diabetes.

### Preoperative laboratory predictors

Some predictors were defined specifically for this study. Among the preoperative laboratories, urinary protein was considered positive for those with any level of protein identified. The average hemoglobin, including the preoperative and first three postoperative values, had a larger effect size than the preoperative value alone and was used as a predictor. Severe hyperglycemia was defined as any glucose value ≥ 180 mg/dl. Hypoglycemia was defined as glucose < 54 mg/dl. HgA1C and serum albumin were also assessed. The preoperative WBC was included to determine if there was any difference in predicting AKI in comparison with the postoperative day POD #1 WBC.

### Procedure-related predictors

In procedure-related characteristics, minimally invasive surgery was laparoscopic, with some robotic or robot-assisted cases included. Ureteral stents included those with unilateral or bilateral stent placement for ureter identification. Bolus therapy with either Ringer’s lactate (RLB) or normal saline (NSB) was identified separately from continuous infusions and was considered as a binary variable. Transfusion volume was the volume of blood given per case. Any patient with net inputs and outputs (I & O’s) between 0 and 3 L on the morning after surgery was euvolemic.

### Procedure related laboratories

#### Clinical markers of inflammation

Among the procedure-related laboratories, the WBC has two important characteristics: it is common and can be obtained at varying perioperative phases, which could exhibit different levels of inflammation. The POD #1 WBC was obtained in 82% of patients and reflected some elements of septic and aseptic inflammation, since 12.8% of patients presented with a preoperative infection.

Neutrophil-to-lymphocyte ratios (NLR) on POD # 1 were less frequently available (40.0%), but may be a more sensitive predictor for complications in patients with aseptic inflammation [16]. The NLR is less useful in patients with sepsis. The NLR at this time would not be expected to reflect a postoperative infection, since there was little time for such an infection to develop. Consequently, the POD # 1 WBC was used as a marker of inflammation for all colorectal patients, including those with sepsis, and the POD # 1 NLR was used as a marker for patients with aseptic inflammation only. It would also most likely represent peak inflammation since it was obtained within 24 h of surgery.

Procedure duration was used as an indirect marker of inflammation, as more extensive dissection, which would increase aseptic inflammation, is associated with longer operative time and surgical intensity [15, 17]. Specific markers of inflammation, such as C-reactive protein, or cytokines were not available in this retrospective study.

### Medications

Medications were considered as either given or not given in the first three postoperative days. Dosages were not evaluated. Perioperative steroids were used to control postoperative nausea. Angiotensin-converting enzyme (ACE) inhibitors and angiotensin receptor blockers (ARB) are often held postoperatively for 48 h because of a link to AKI. The holding of ACE/ARBs for patients on these medications was evaluated as a predictor.

A large variety of predictors were chosen so that the relative importance of inflammation in inducing AKI could be evaluated. Only predictors that were reliably included in the electronic medical record and identified in Epic electronic health records (Verona, WI) were assessed. Not all information related to AKI could be evaluated; for instance, intraoperative fluid administration could not be determined, and the length and degree of perioperative hypotension, although important, was beyond the scope of this retrospective review.

### Secondary outcomes

Perioperative complications included postoperative infections, chronic renal insufficiency (CRI), non-infectious complications, 30-day readmissions, and length of stay (LOS). Infections were calculated by documenting positive clinical cultures in the postoperative period from Day 1 to Day 30, as previously described [18]. Cultures were considered positive if pathogenic bacteria were identified, not normal flora. Mixed urogenital flora, or normal skin flora were not considered positive. A patient was considered to have a positive culture if one or more cultures were positive.

Chronic renal insufficiency was defined as an increase in creatinine of ≥ 0.3 mg/dl over the baseline creatinine from 3 to 12 months after surgery. This numerically small increase in creatinine was chosen as a cutoff because of the documented increase in complications associated with the earliest stage of AKI.

A variety of significant non-infectious in-hospital complications were also included and were extracted with software from Conduent-Midas Health Analytics Solutions (Florham Park, NJ). Respiratory and cardiopulmonary failure or arrest, acute myocardial infarction or stroke, and deep vein thrombosis or pulmonary embolus were included. Infectious and non-infectious complications were combined for an in-hospital complication rate. For combined complications, any patient was considered positive with one or more complications.

### Data interpretation

Data was electronically extracted from Epic and analyzed using Excel. Data for continuous variables was expressed as mean and standard deviation; categorical variables were expressed as percentages. Two sample t-tests and tests for two proportions were used to compare groups. Mann-Whitney non-parametric tests were used for non-normally distributed data. Univariate logistic regression was used to determine significant (*P* < 0.05) associations between potential predictors and AKI, and multivariate regression with backward elimination was employed to determine independent predictors.

### Data availability

The datasets generated or analyzed during the current study are not publicly available since they are private health information, but the data could be made available from the corresponding author upon reasonable request, consistent with IRB review and requirements.

## Results

### Primary outcome

AKI occurred in 24.6% of patients. The demographic information for those who did or did not develop AKI is listed in Table 1.

**Table 1:** Demographics in patients who did or did not develop AKI

AKI	Patients	Male	Age	BMI	History of DM	ASA
No	938	438 (46.7%)	63.8 ± 14.8	29.04 ± 6.6	160 (17.1%)	2.89 ± 0.7
Yes	286	159 (55.6%)	65.7 ± 13.7	30.2 ± 8.3	64 (22.4%)	3.09 ± 0.7
P Value		0.010	0.048	0.016	0.064	< 0.0001

Patients who developed AKI were significantly older, more likely male, had higher BMI and ASA scores, and a tendency towards diabetes.

The increased co-morbidity status of patients with AKI is also seen in the preoperative laboratory values, with a significantly lower mean hemoglobin and albumin concentration, and an increased creatinine, as seen in Table 2. There was no difference in the preoperative WBC between those who did or did not develop AKI. Patients with an active infection at the time of surgery had a higher POD #1 WBC (15.23 ± 7.3) than those without infection (12.64 ± 5.1, *P* < 0.0001), but there was no difference in the rate of AKI (26.3% in patients with a preoperative infection, 22.9% without infection, *P* = 0.428).

**Table 2:** Preoperative laboratories

AKI	Patients	Hg	Creatinine	Albumin	WBC
No	938	12.23 ± 2.17	1.04 ± 0.66	3.13 ± 0.73	9.59 ± 5.92
Yes	286	11.60 ± 2.29	1.16 ± 0.90	2.85 ± 0.76	9.48 ± 5.35
P Value		0.0001	0.020	0.0001	0.776

The surgical approach did not appear to be related to those who developed AKI. Depicted in Table 3 are three procedure-related characteristics, open versus minimally invasive approach, emergent versus elective, or the use of ureteral stents for ureter identification, which did not differ in patients with or without AKI.

**Table 3:** Procedure-related characteristics

AKI	Patients	Laparoscopic	Emergent	Urinary Stents
No	938	450 (48.0%)	193 (20.6%)	307 (32.7%)
Yes	286	121 (42.3%)	66 (23.1%)	100 (35.0%)
P Value		0.104	0.420	0.532

### Univariate predictors of AKI

**Fig. 1:**
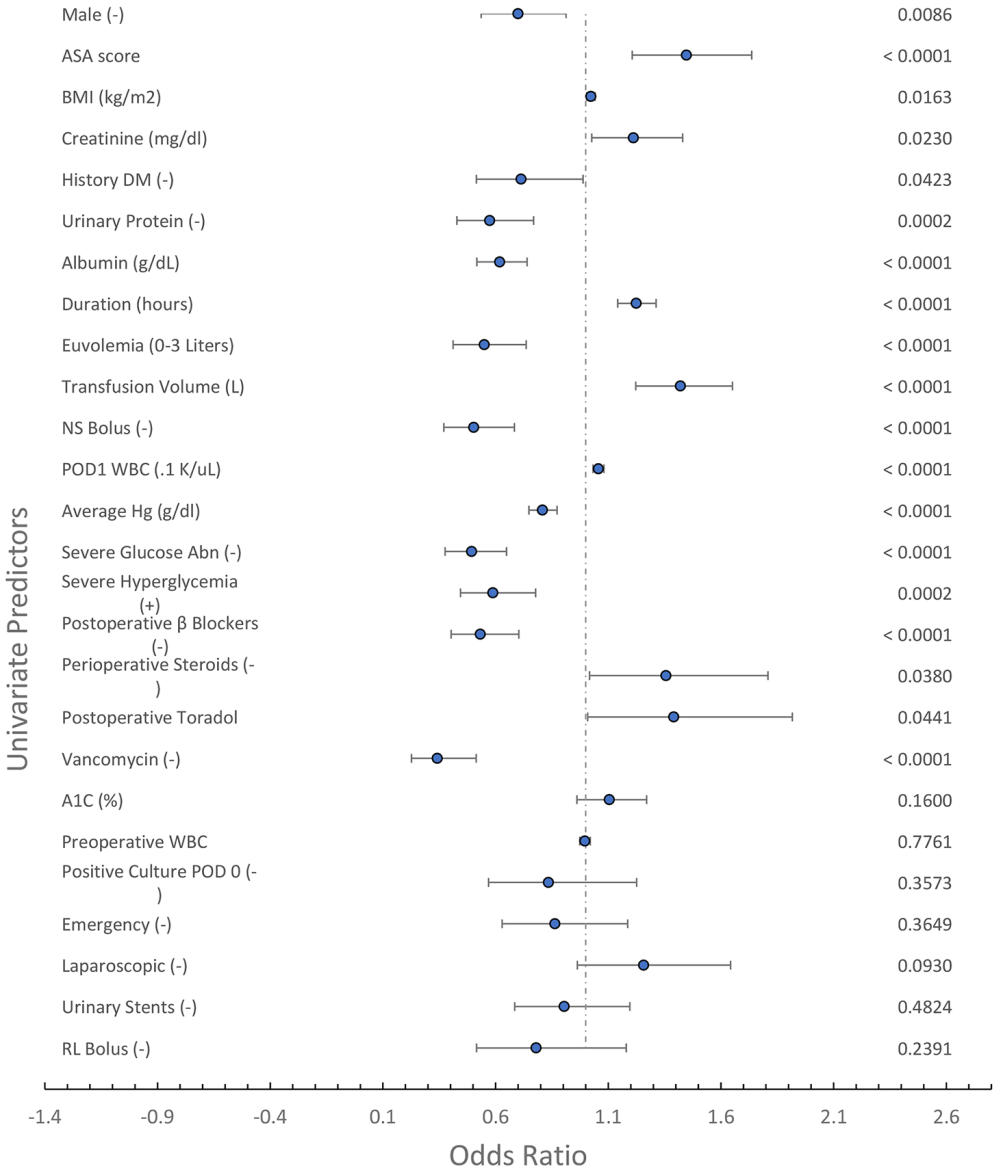
Forest plot of univariate predictors of AKI. Significant (*P* < 0.05) univariate predictors of AKI are depicted. The POD #1 WBC, procedure duration, and the use of ketorolac, and steroids correlate with systemic inflammation. Some predictors of interest without association with AKI are listed near the bottom of the Forest Plot. Euvolemia: a patient with a positive fluid balance between 0 and 3 L on the morning after surgery. Severe Glucose Abnormality: either severe postoperative hyperglycemia (glucose > = 180 mg/dl), or severe hypoglycemia (glucose < 54 mg/dl). Pos Cul POD 0: a patient with a positive culture on the day of surgery. NS or RL bolus: bolus fluid therapy of either normal saline or Ringer’s lactate. DF: The univariate analyses were based on 1224 patients, with a few missing parameters for each predictor, except the following: HgA1C: DF: 690

The univariate predictors of AKI are seen in Fig. 1. Twenty predictors with a P value < 0.05 on logistic regression are included, with 7 selected non-significant predictors of interest. The significant predictors are on the top part of the Forest Plot, with the non-significant predictors of interest on the bottom. Most of the predictors of AKI seen here have been previously reported and are consistent with the literature.

The most direct marker of inflammation– the POD #1 WBC– was among the predictors with the largest effect size for AKI. Aside from the POD # 1 WBC, procedure duration is considered a measure of surgical stress and was also significantly predictive of AKI (*P* < 0.0001). Finally, two medications, each with anti-inflammatory properties, were associated with a lower AKI: steroids and non-steroidal anti-inflammatory drugs (NSAIDs).

### Multivariable analysis

**Fig. 2:**
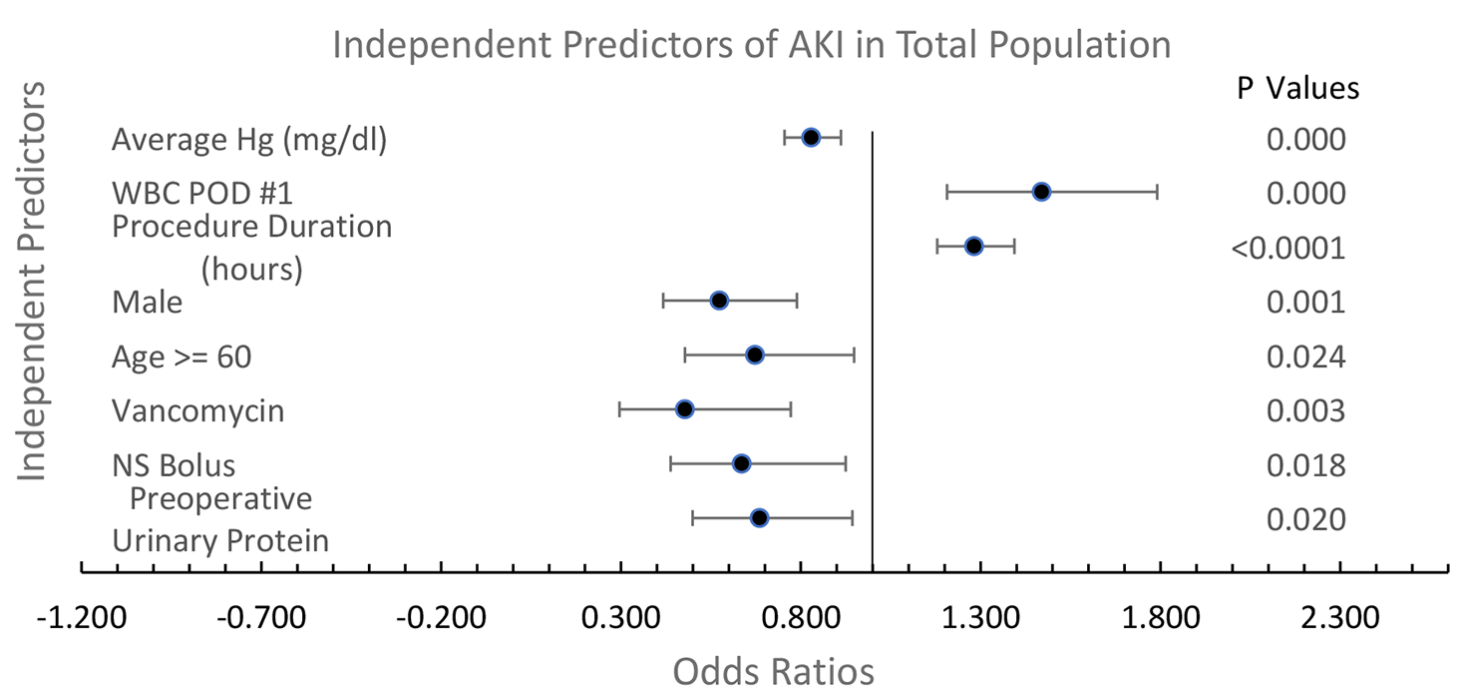
Multivariable predictors of AKI of total population. Factors associated with systemic inflammation are independently associated with AKI. AUC: 0.73; DF: 982

Multivariable analysis of the total population of patients showed that two demographic parameters (sex and age), 1 peri-procedural laboratory value (average Hg), 2 peri-procedural characteristics (transfusion volume and administration of NS bolus), 1 medication (vancomycin), and two factors related to inflammation (procedure duration, and POD #1 WBC) were independent predictors of AKI. The multivariable analysis is depicted in Fig. 2.

### Predictors of AKI in aseptic population

#### Univariate predictors of AKI in aseptic patients

The relationship between AKI and the univariate variables was examined in the subpopulation of patients with septic inflammation. The POD # 1 NLR was significantly associated with AKI (*P* = 0.000), as was procedure duration (P < < 0.0001). The other univariate predictors were similar to the total population (data not shown).

#### Multivariate predictors of AKI in aseptic patients

**Fig. 3:**
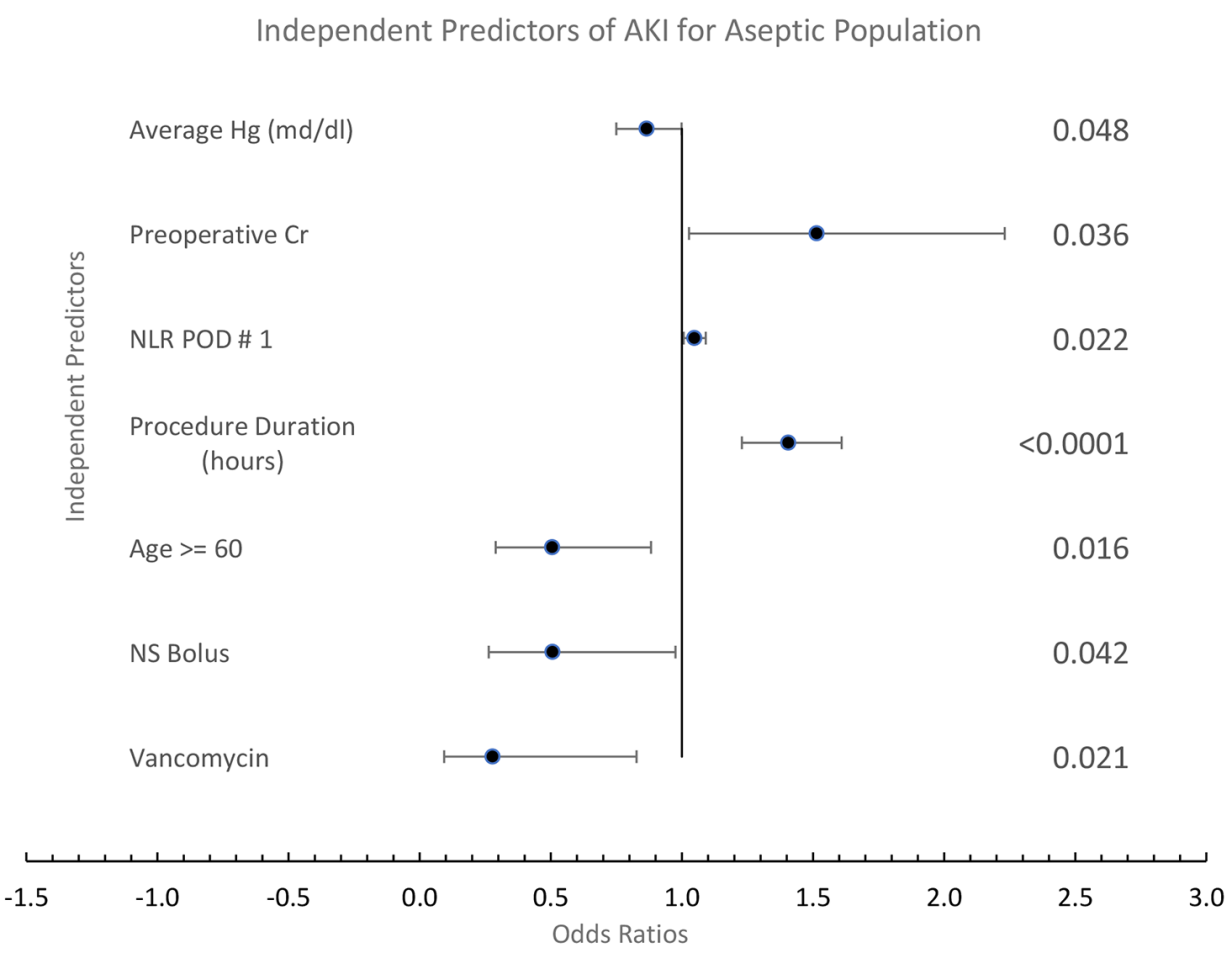
Multivariable predictors of AKI of aseptic population. Cr: creatinine. AUC: 0.751; DF: 468

Similar findings were evident in patients without sepsis at the time of surgery. Fifteen significant (*P* < 0.05) univariate predictors were placed in a multivariable model. Even in the absence of sepsis, variables related to inflammation, the POD # 1 NLR (*P* = 0.022), and procedure duration (*P* < 0.0001) were among 7 independent predictors of AKI, as seen in Fig. 3.

### Perioperative complications

**Fig. 4:**
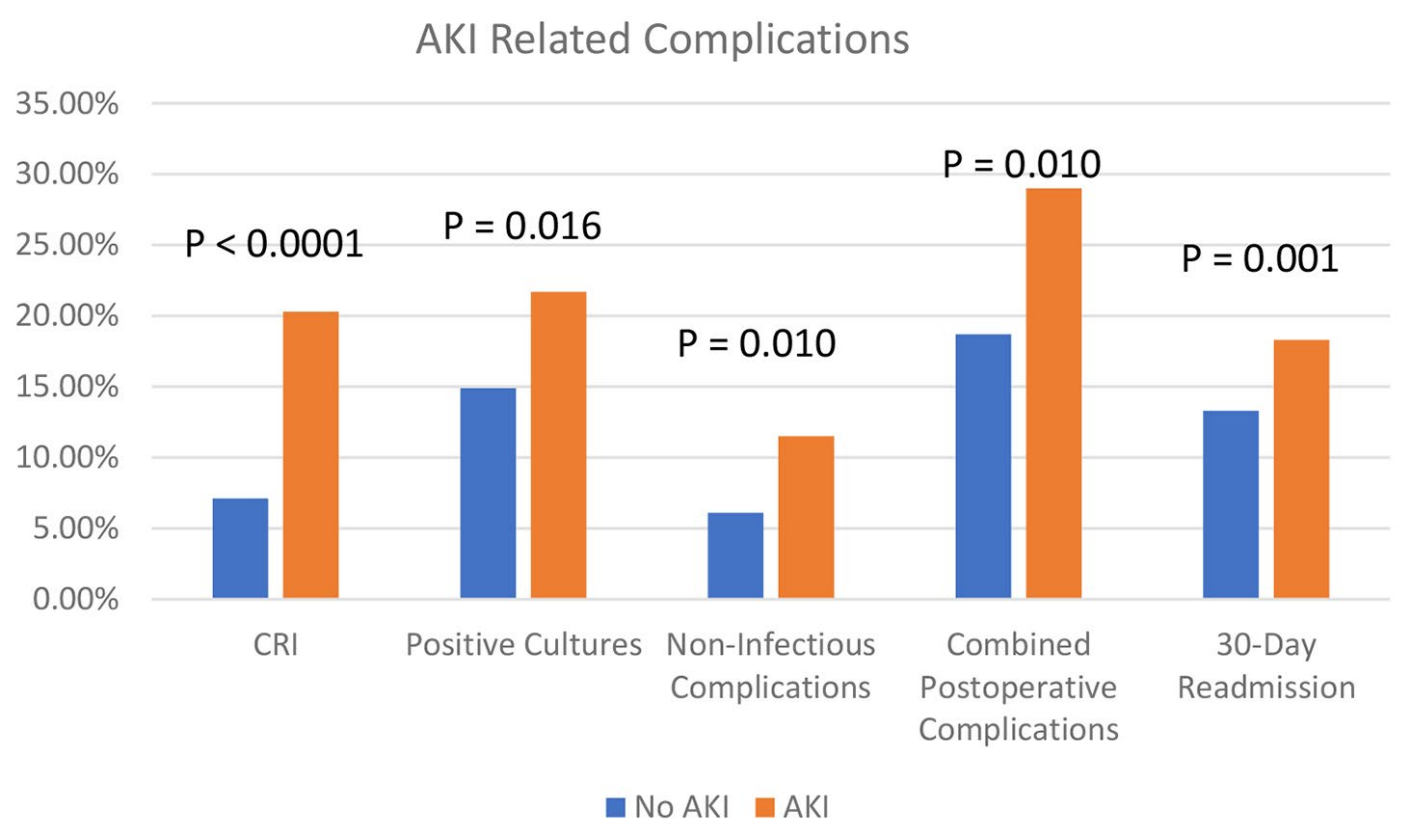
Complications in patients with or without AKI. Chronic renal insufficiency (CRI) was assessed between 3 months and 1 year after surgery. Infectious complications occurred within 30 days of surgery. Non-infectious complications occurred during the index surgical hospitalization. Combined complications included infectious and non-infectious complications. The data was derived from 1224 patients, except for the long-term creatinine, which was available in 829 patients

Patients who developed AKI had a significantly higher complication rate in all categories, with a greater percentage of patients with CRI, infections, non-infectious complications, combined in-hospital complications, and 30-day readmissions. The difference in complications in patients with and without AKI is seen in Fig. 4.

For the total population of colorectal patients, the most common marker of postoperative systemic inflammation– the POD # 1 WBC– was associated with multiple postoperative complications on logistic regression: infections (*P* = 0.002), non-infectious complications (*P* = 0.012), in-hospital complications (*P* = 0.000), 30-day readmissions (*P* = 0.027), and LOS (P = < 0.0001), as seen in Table 4. The LOS was significantly different (*P* < 0.0001) between those with (17.2 ± 25.6) or without (9.5 ± 11.8) AKI.

**Table 4:** POD # 1 WBC as a predictor of outcomes in total population

Logistic Regression					
Outcome	N	OR	Lower Bound	Upper Bound	P Value
AKI	990	1.056	1.032	1.081	< 0.0001
Postoperative Infection	990	1.040	1.014	1.066	0.002
Non-Infectious Complications	990	1.040	1.009	1.072	0.012
In-Hospital Complications	990	1.044	1.020	1.069	0.000
30-Day Readmissions	990	1.031	1.004	1.060	0.012
Linear Regression					
Outcome	N	Coefficient	Upper Bound	Lower Bound	P Value
LOS	990	0.495	0.314	0.677	< 0.0001

In patients with aseptic inflammation, the POD # 1 NLR was significantly associated with postoperative infections (*P* = 0.008), in-hospital complications (*P* = 0.002), and LOS (*P* = 0.006), as seen in Table 5.

**Table 5:** NLR* as a predictor of outcomes in aseptic population

Logistic Regression					
Outcome	N	OR	Lower Bound	Upper Bound	P Value
AKI	485	1.073	1.034	1.113	0.000
Postoperative Infection	485	1.062	1.016	1.111	0.008
Non-Infectious Complications	485	1.044	0.985	1.107	0.148
In-Hospital Complications	485	1.066	1.024	1.109	0.002
30-Day Readmissions	485	1.021	0.976	1.069	0.371
Linear Regression					
Outcome	N	Coefficient	Lower Bound	Upper Bound	P Value
LOS	485	0.467	0.136	0.797	0.006

*: neutrophil-to-lymphocyte ratio

## Discussion

AKI, even in the earliest stage, or with mild elevations not even reaching stage 1 [19], is associated with serious peri-procedural and early to mid-term complications. Increased rates of infection [20], a progression to chronic renal disease [21], and worsening of 90-day [22] and two-year mortality [23] have been most commonly documented. Numerous studies have shown that the causes of postsurgical AKI are multiple, and some have been modifiable; yet there have been no studies that have shown that avoiding AKI can lower the complication rate, possibly related to the complexity of its origin. It is hoped that a more complete understanding of the multiple causes of AKI can lead to clinical progress.

Recent studies have highlighted an interaction between components of the inflammatory cascade and AKI, both for aseptic and septic etiologies [24]. Damage-associated molecules (DAMPS) are released from necrotic cells, and pathogen-associated fragments (PAMPS) are released from pathogens that activate multiple cytokine pathways, which in turn engage the immune system. Neutrophils and early-stage macrophages are attracted to the kidney, which have received a variety of insults, and add to the peri-renal inflammatory process. Kidney damage reduces cytokine clearance, disturbing the typical inflammatory resolution, and leads to a paralysis of some components of the inflammatory mechanism, such as the recruitment of WBCs. The net result is increased infections associated with AKI, even in non-septic etiologies. Systemic inflammation can add to local renal damage induced by other injury mechanisms, and the renal injury exacerbates and prolongs a systemic inflammatory state.

The predictors of post-surgical AKI are multiple, varied, and remarkably consistent. No one marker explains a large percentage of the cases; rather, AKI results from an incremental addition of insults. Age, sex, hemoglobin concentration, preoperative renal dysfunction, nephrotoxic medication, the administration and composition of peri-operative fluids, and hemodynamic instability are reported with regularity as causes of AKI [1, 7, 25–28], and are evident in this study. Systemic inflammation may be an equally important etiologic factor. It is not clear if some of these conditions, such as age or BMI, might assert their injury through an inflammatory mechanism [29].

In this retrospective review, no direct measures of inflammation were available, but the POD # 1 WBC and NLR and procedure duration were chosen as available, clinically useful markers. Inflammation peaks early after surgery, with interleukin-6 levels highest on POD #1, and CRP levels highest on POD 2–3 [30, 31]. The importance of inflammatory indices early after surgery has been suggested by several studies. POD # 1 NLR was predictive of AKI after cardiac surgery [32]. In patients who underwent noncardiac surgery after drug-eluting stent placement, NLR on POD # 1 was associated with cardiac-related complications [33]. Systemic inflammatory index (SII), assessed 1 h after admission to the ICU, was associated with severe complications after upper abdominal surgery [34]. Our results confirm the relationship between inflammatory indices within 24 hours of surgery and complications.

The strongest predictor of AKI was surgical duration. An operation may be longer because of hemodynamic instability and fluid shifts, which are non-inflammatory causes of AKI, but is also lengthened by the extent and difficulty of the surgical dissection, which increases inflammation. The WBC is a recognized marker of inflammation, and it is usually used to estimate the severity of infection. In our study, the POD # 1 WBC reflected both septic and aseptic inflammation for all colon patients and was associated with AKI and other outcomes. The NLR may be a more appropriate marker of inflammation in patients without sepsis, and the NLR confirms the association between aseptic inflammation and outcomes. Aseptic inflammation may be an important cause of AKI and subsequent complications.

Aseptic inflammation is an inevitable and necessary response to surgery. However, inflammation marked by AKI may exceed the requirement for ideal healing after surgery. AKI may be a predecessor for a prolonged, unneeded inflammatory state. A beneficial initial inflammation might be followed by inflammation-induced complications [20]. The variety and time course of complications associated with small elevations of creatinine supports the view that a systemic process, inflammation, might be the instrument that induces the complications. The association of anti-inflammatory interventions with a lower AKI raises the possibility that inflammation-related AKI may be modifiable. However, anti-inflammatory strategies would have to be carefully considered, as there is the potential for adverse events [35]. The extent to which the pro-inflammatory and anti-inflammatory pathways can be manipulated is uncertain.

## Limitations

This is a retrospective review, and the data collected do not represent randomized samples. We can only note a correlation, not a causation, between inflammation and AKI and AKI-related complications. It is not possible to determine the influence of good clinical judgment on the role of the predictors of AKI. In addition, not every important cause of AKI could be extracted from the electronic record. Intraoperative fluid management was not available, and perioperative hemodynamic information was not reviewed as it was not readily extractable. The retrospective review makes it difficult to propose a causal relationship between specific risk factors and AKI but serves as hypothesis generating. Finally, indirect rather than direct markers of inflammation were studied.

Many factors that are highly significant predictors of AKI on univariate analysis lost significance with multivariable review. Preoperative albumin levels and peri-operative glucose control have been reported as independent predictors of AKI. These predictors may have been tightly associated with other factors, so a larger sample size, or different cohort of patients, may reveal their independent importance.

## Conclusions

The etiologies of AKI are diverse and numerous after colorectal surgery. Systemic inflammation plays a significant role in the pathogenesis and the consequences of AKI. Limiting excessive inflammation without interfering with the healing process might reduce postoperative AKI and improve outcomes.

## Data Availability

The datasets generated or analyzed during the current study are not publicly available since they are derived from private health information, but the non-identifiable summaries of the data could be made available from the corresponding author upon reasonable request, consistent with IRB review and requirements.

## Declarations

### Meeting presentation

This work has not yet been presented to any meeting.

### Precis

Benign-appearing increases in creatinine after colorectal surgery are associated with complications that are wide-ranging, often delayed, and serious. Systemic inflammation, more than what is needed for healing, could be the cause. In 1224 consecutive colorectal operations, parameters associated with inflammation were independently associated with AKI and AKI-related complications.

## Abbreviations

AKI: Acute Kidney Injury
KDIGO: Kidney Disease Improving Global Outcomes
POD # 1 WBC: The first postoperative white blood cell on postoperative day 1
POD # 1 NLR: The neutrophil-to-lymphocyte ratio on postoperative day 1
CRI: Chronic Renal Insufficiency
ASA: American Society of Anesthesia
RLB: Ringer’s lactate
NSB: Normal Saline Bolus
BMI: Body Mass Index
LOS: Length of Stay
NSAIDs: Non-steroidal anti-inflammatory medications
